# Extensive clinical, hormonal and genetic screening in a large consecutive series of 46,XY neonates and infants with atypical sexual development

**DOI:** 10.1186/s13023-014-0209-2

**Published:** 2014-12-14

**Authors:** Dorien Baetens, Wilhelm Mladenov, Barbara Delle Chiaie, Björn Menten, An Desloovere, Violeta Iotova, Bert Callewaert, Erik Van Laecke, Piet Hoebeke, Elfride De Baere, Martine Cools

**Affiliations:** Center for Medical Genetics, Ghent University Hospital and Ghent University, Ghent, Belgium; Department of Pediatric Endocrinology, Ghent University Hospital and Ghent University, Building 3K12D, De Pintelaan 185, 9000 Ghent, Belgium; Department of Pediatrics and Medical Genetics, Medical University of Varna, University Hospital “Sveta Marina”, Varna, Bulgaria; Department of Pediatric Urology, Ghent University Hospital and Ghent University, Ghent, Belgium

**Keywords:** 46,XY DSD, Undervirilization, Integrated screening, Diagnosis, Array-CGH, MLPA, *NR5A1* mutations

## Abstract

**Background:**

One in 4500 children is born with ambiguous genitalia, milder phenotypes occur in one in 300 newborns. Conventional time-consuming hormonal and genetic work-up provides a genetic diagnosis in around 20-40% of 46,XY cases with ambiguous genitalia. All others remain without a definitive diagnosis. The investigation of milder cases, as suggested by recent reports remains controversial.

**Methods:**

Integrated clinical, hormonal and genetic screening was performed in a sequential series of 46, XY children, sex-assigned male, who were referred to our pediatric endocrine service for atypical genitalia (2007–2013).

**Results:**

A consecutive cohort of undervirilized 46,XY children with external masculinization score (EMS) 2–12, was extensively investigated. In four patients, a clinical diagnosis of Kallmann syndrome or Mowat-Wilson syndrome was made and genetically supported in 2/3 and 1/1 cases respectively. Hormonal data were suggestive of a (dihydro)testosterone biosynthesis disorder in four cases, however no *HSD17B3* or *SRD5A2* mutations were found. Array-CGH revealed a causal structural variation in 2/6 syndromic patients. In addition, three novel *NR5A1* mutations were found in non-syndromic patients. Interestingly, one mutation was present in a fertile male, underlining the inter- and intrafamilial phenotypic variability of *NR5A1*-associated phenotypes. No *AR*, *SRY* or *WT1* mutations were identified.

**Conclusion:**

Overall, a genetic diagnosis could be established in 19% of non-syndromic and 33% of syndromic cases. There is no difference in diagnostic yield between patients with more or less pronounced phenotypes, as expressed by the external masculinisation score (EMS). The clinical utility of array-CGH is high in syndromic cases. Finally, a sequential gene-by-gene approach is time-consuming, expensive and inefficient. Given the low yield and high expense of Sanger sequencing, we anticipate that massively parallel sequencing of gene panels and whole exome sequencing hold promise for genetic diagnosis of 46,XY DSD boys with an undervirilized phenotype.

**Electronic supplementary material:**

The online version of this article (doi:10.1186/s13023-014-0209-2) contains supplementary material, which is available to authorized users.

## Background

The birth of a child with ambiguous genitalia is a rare event with a prevalence of one in 4500 live births and poses a challenge to the parents and the medical team [1]. Specialized multidisciplinary medical care, aiming at addressing concerns and uncertainties with regard to gender assignment, underlying etiology and management, as well as providing adequate psychological support is essential [2]. Extensive and time-consuming hormonal and genetic work-up provides a genetic diagnosis in 20-40% of cases [3,4]. Less pronounced atypical development of male external genitalia is more prevalent and is noticed in the newborn period in around one in 300 males; 75% of cases are associated with hypospadias [5]. These milder forms of undervirilization, such as isolated or combined cryptorchidism and hypospadias have been related to environmental factors, low birth weight and multiple gene polymorphisms rather than single gene mutations [6-8]. However, mutations in the *Androgen Receptor (AR)*, *Nuclear Receptor Subfamily 5 Group A Member 1 (NR5A1)* and *Wilms Tumor 1 (WT1)* genes - classically associated with genital ambiguity or more severe forms of undervirilization - have recently been identified in cases with isolated proximal or even distal hypospadias, combined cryptorchidism and (distal) hypospadias or anorchia, and sequencing of these genes has been advocated in such cases [9-14]. On the other hand, copy number variations in genes involved in the process of sexual development have effectively been detected by whole genome (array comparative genomic hybridization, array-CGH) or targeted (multiplex ligation-dependent probe amplification, MLPA) copy number analysis [15-17], and both techniques have become widely available in recent years. However, whether a systematic extensive genetic work-up is indicated in the 46,XY newborn with a milder degree of undervirilization, as indicated by a higher Prader or External Masculinization Score (EMS) remains a matter of debate [18]. Current screening methods are time consuming and have a low efficiency. The introduction of genome-wide technologies such as whole exome sequencing (WES) holds promise for future clinical decision making in a routine diagnostic setting for these rare, genetically heterogeneous conditions.

In order to gain insight in the appropriateness and diagnostic yield of a systematic genetic work-up in 46, XY infants with atypical external genitalia, we performed a standardized genetic screening panel in all 46,XY neonates and infants who were referred to our pediatric endocrine service for atypical male or ambiguous genitalia in the period 2007–2013 and who received male sex assignment. This screening consisted of consecutive Sanger sequencing of the *AR, NR5A1* and *WT1* genes, high-resolution (180 K) array-CGH and a commercially available MLPA kit with probes for *Sex Determining Region Y* (*SRY*), *SRY-box 9* (*SOX9*)*, Nuclear Receptor Subfamily 0 Group B Member 1* (*NR0B1*)*, Wingless type 4* (*WNT4*) and *NR5A1.* Additionally, sequencing of *SRY* was performed in cases with hormonal results consistent with the presence of (partial) gonadal dysgenesis, and sequencing of *Hydroxysteroid (17-Beta) Dehydrogenase (HSD17B3*) or *Steroid-5-Alpha-Reductase, Alpha Polypeptide 2* (*SRD5A2*) was performed in cases with suspicion of a (dihydro)testosterone biosynthesis defect. Results were interpreted in the light of clinical and hormonal findings.

## Patients and methods

### Patients

All 46,XY children younger than two years who were referred to our pediatric endocrinology service for the evaluation of atypical genitalia (e.g. hypospadias, micropenis) and who were sex assigned male, between 2007–2013 were included (n = 32) (Table 1). Medical history included pregnancy details, birth weight (BW), consanguinity and a familial history of disorders of sex development (DSD), sub- or infertility, premature ovarian failure (POF) or atypical genitalia. Phenotypic description consisted of a physical examination with special attention to dysmorphism; EMS scores were calculated based on the aspect of the external genitalia [19]. None of the patients had proteinuria or renal insufficiency.

**Table 1:** **Medical history and phenotypic details of patients**

**Code**	**GA (weeks)**	**BW (g)**	**BW (SD)**	**EMS**	**Pregnancy**	**Dysmorphic features**	**Consanguinity**	**Family history**
1	32	900	−3.15	2/12	CS	IUGR	No	Unremarkable
2	41	3.260	−1.12	3/12	Normal	Large ears	No	Unremarkable
						Broad nose		
						Mild frontal bossing		
3	40	4.150	1.12	2,5/12	Normal	/	No	Maternal aunt: difficulties to get pregnant, one child with congenital abnormalities
4	41	3.060	−1.6	3/12	Normal	/	No	Unremarkable
					Minoxidil treatment			
5	34	1.320	−2.93	7,5/12	CS	IUGR	No	Unremarkable
6	40	3.380	−0.59	9/12	Normal	/	No	Grandfather with hypospadias, maternal aunt with POF
7	30	510	−3.8	6/12	Induced delivery	IUGR	No	Paternal grandmother: cleft lip
					Twins			
8	39	3.690	0.45	10/12	IVF	/	No	Unremarkable
9	38	3.310	0.01	8/12	Normal	/	No	Unremarkable
10	39	2.850	−1.53	9/12	Placental infarction	Microcephaly	No	Unremarkable
						Mild facial dysmorphism		
11	40	3.000	−1.51	3/12	Normal	Macrocephaly	No	Unremarkable
						Facial dysmorphism		
						Short neck		
						Developmental delay (speech)		
12	40	3.850	0.47	6/12	Normal	/	Yes	Mother: fertility problems, irregular menses
13	35	2.530	−0.26	6/12	Preeclampsia and hypertension	/	No	Unremarkable
					Obesity			
					Twins			
					Sectio			
14	32	945	−2.98	3/12	Bleeding	/	No	Unremarkable
					CS			
15	40	NA	NA	3/12	Normal	/	No	Unremarkable
16	28	860	−1.26	7/12	Eclampsy	Atrial septum defect	No	Unremarkable
					Prematurity			
17	34	1.450	−2.52	6/12	CS	Ventricular septum defect	Yes	Cousin (deceased)with Jeune syndrome
18	34	1.400	−2.68	2/12	CS	/	No	Unremarkable
					Antidepressant Paroxetine			
19	41	2.805	−2.24	3/12	Normal	/	No	Unremarkable
20	39	2.880	−1.45	6/12	Normal	/	Possible	Unremarkable
21	36	1.585	−3.39	7/12	Preeclampsia	/	No	Unremarkable
22	39	2.640	−2.07	6/12	Normal	/	No	Unremarkable
23	40	3.250	−0.9	8/12	Normal	/	No	Unremarkable
24	37	3.290	−0.43	6/12	Normal	/	Yes	Unremarkable
25	38	2.800	−1.20	6/12	Preeclampsia	/	No	Mother: brother deceased from SIDS
								Maternal grandfather: depression
								Maternal grandmother: recurrent miscarriage
								Father: late puberty
								Paternal uncle: retractile testes, normal fertility
26	39	3.770	−0.63	12/12	Preterm contractions	Hypoplastic bulbus olfactorius	/	Unremarkable
27	26	700	−1.26	10/12	IVF	Persisting	/	Unremarkable
					Twins			
					Placental rupture	Periventricular leukcomalacy		
28	36	2.570	−0.72	8/12	Previous abortions	X-linked ichthyosis	/	First child was stillborn
					Preterm contractions	Hypotonia		Three miscarriages between month 1 and 2
						Developmental delay ductus arteriosus		
					Bleeding	Abnormal liver function tests		
29	39	3.230	−0,6	9/12	Pregnancy after gonadotrophin treatment father	/	/	Father with Kallmann syndrome, pregnancy after gonadotrophins
30	38	3.782	1.04	8/12	Normal	Mowat-Wilson syndrome	/	Unremarkable
32	39	2780	−1.70	7/12	Normal	No	No	Unremarkable

GA: gestational age, BW: birth weight, EMS: external masculinization score, IUGR: intra uterine growth retardation, POF: premature ovarian failure, SIDS: sudden infant death syndrome; CS: Caesarian section, IVF: in vitro fertilization.

## Methods

### Biochemical analyses

Hormonal levels were obtained between day 14–90 after birth or after HCG stimulation (Pregnyl®, 1500U, with blood sampling at baseline and after 72 hours). The following hormone levels were measured: anti-Müllerian Hormone (AMH) by enzyme linked immunosorbent assay (Beckman Coulter Company), Androstenedione (A) by Radioimmunoassay (DiaSource Company), Testosterone (T) and Dihydrotestosterone (DHT) by liquid chromatography/tandem mass spectrometry (UPLC Waters quattro premier). LH and FSH by electrochemoluminescence assay (Roche Diagnostics E170 Modular).

#### Genetic analyses

Array-CGH using the Agilent 180 K array was used as a genomewide screen for copy number variations (CNVs) with an overall mean probe spacing of 14 kb, or 11 kb when only taking into account the Refseq genes. Hybridization was done according to the manufacturer’s protocol, followed by visualization of the results in arrayCGHbase [20]. Fluorescent in situ hybridization (FISH) was performed for *SRY* to search for *SRY* rearranging translocations and mosaicism. To screen for CNVs on the exon level, MLPA was done using the SALSA MLPA P185 Intersex probemix (MRC-Holland) containing probes for *NR0B1, NR5A1, SOX9, SRY* and *WNT4*. Sanger sequencing of the coding exons and untranslated regions (UTRs) was used to identify mutations in *AR, NR5A1 and WT1. SRY* sequencing was included for patients suspected to have gonadal dysgenesis, based on an AMH level below the reference range. *HSD17B3* and *SRD5A2* were sequenced in cases with suspicion of a testosterone biosynthesis disorder based on a T/A ratio <1 for 17β-HSD deficiency and a T/DHT ratio > 8.5 for 5α Reductase Deficiency (Table 2) [21,22]. Primers for *AR*, *WT1* en *SRY* were designed using PrimerXL (http://www.primerxl.org/, available on request). Primer sequences for *NR5A1*, *HSD17B3* and *SRD5A2* can be found in supplemental data (Additional file 1: Table S1). *Zinc Finger E-Box Binding Homeobox 2* (*ZEB2*) sequencing and sequencing of the Kallmann syndrome (KS) gene panel, consisting of six genes (*KAL1*, *CHD7*, *FGFR1*, *PROK2*, *PROKR2*, *FGF8*) was done at the Henri Mondor Hospital (Paris, France). *Fibroblast Growth Factor Receptor 1* (*FGFR1*) sequencing was performed at the CHU Hospital Cochin (Paris, France).

**Table 2:** **Hormonal and genetic data of patients**

**Code**	**FSH (U/l)# (ref)**	**AMH (** **μ** **g/l) (ref)$**	T (ng/dl)*/**	***AR***	***NR5A1***	***WT1***	***SRY***	**Array-CGH**	**MLPA**	**Other**
1	3.2(1–12)	59.8(46.8-173)	136*	Nl	Nl	Nl	FISH Nl	Nl	Nl	*HSD17B3*
2	14.0(1–12)	62.7(46.8-173)	63.8*/579**	/	Nl	Nl	Nl	Nl	Nl	*HSD17B3*, *ATRX* N
3	6.6(1–12)	10.8(105–270)	4.7*	/	c.253_254del	/	Nl	/	/	
4	1.6(1–12)	118(46.8-173)	195*	Nl	Nl	Nl	/	Nl	Nl	
5	NA	152(67.4-197)	526**	Nl	c.437G > C (tolerated)	/	FISH Nl	/	/	
6	3.3(1–12)	64.5(62–130)	184*	Nl	c.630_637del	/	FISH Nl	/	Nl	
7	1.5(1–12)	57.6(105–270)	222*	Nl	Nl	Nl	/	7q36.3q36.3 (158189154–158343770)×1, maternal	Nl	
8	NA	10.6(38–180)	275**	Nl	Nl	Nl	Nl	Nl	Nl	
9	NA	231(46.8-173)	NA	Nl	Nl	Nl	/	Xp22.33p22.33 (839417–1179089)×3, maternal	Nl	
10	NA	244(46.8-173)	NA	Nl	Nl	Nl	/	Nl	Nl	*ATRX* Nl
11	5.5(1–12)	NA	158*/511**	/	/	/	/	3p25(RP11-385A18 → RP11-334 L22)×3, 9p24.3 (RP11-48 M17 → RP11-320E16)×1	/	
12	2.5(1–12)	147(46 · 8-173)	109*	/	Nl	Nl	/	/	Nl	
13	NA	72.7(46.8-173)	280*	/	Nl	Nl	FISH Nl	Nl	Nl	
14	1.2(1–12)	8.6(105–270)	248*	Nl	/	/	/	/	/	
15	NA	132.9(42–185)	NA	Nl	c.1109 G > A (p Cys370Tyr)	/	FISH Nl	16p12.3p12.3 (18894303–19162153)×3,maternal (normal variant)	/	
16	1.3(1–12)	298(67.4-197)	337*	Nl	Nl	Nl	FISH Nl	Xq13.3q13.3 (74285912–75325119)×2, maternal	Nl	
17	NA	72(38–180)	NA	/	Nl	Nl	FISH Nl	/	Nl	*HSD17B3*
18	2.6(1–12)	82.2(23.8-124)	NA	Nl	Nl	Nl	/	2p16.3p16.3 (5073244–50894316)x1; 16p13.11p13.11(15830681–16270149)x3	Nl	
19	1.8	49.1(23.8-124)	35.3*/390**	Nl	Nl	Nl	FISH Nl	Xq13.3q13.3(74380482–74567915)×2, maternal	Nl	
20	2.8(1–12)	194(105–270)	104*	Nl	Nl	Nl	FISH Nl	Nl	Nl	
21	1.9(1–12)	28.8(55.3-187)	NA	Nl	Nl	Nl	Nl	/	Nl	
22	2.1(1–12)	94.1(105–270)	151*	Nl	Nl	Nl	Nl	5p14.3p14.3(21438696–21490654)×1, maternal, 14q21.2q21.3(42908541–43293564)×3, maternal	Nl	
23	NA	11.27(55.3-187)	404**	Nl	Nl	Nl	Nl	Nl	Nl	
24	NA	43(105–270)	152*/474**	/	Nl	Nl	Nl	Nl	Nl	*SRD5A2* N
25	1.0(1–12)	156(105–270)	207.3*	Nl	Nl	Nl	/	Nl	Nl	
26	0.17(1–12)	65.9(105–270)	3.3*/90.8 **	/	/	/	/	Nl	/	
27	NA	245(55.3-187)	951**	Nl	Nl	Nl	/	/	Nl	
28	NA	14.03(55.3-187)	NA	/	/	/	/	Xp22.32p22.31(5405569–9222059)×0, maternal	/	
29	0.57(1–12)	NA	3.2*	/	/	/	/	/	/	*FGFR1* c.1042G > A
30	1.2(1–12)	159(105–270)	502*	/	/	/	/	Nl	/	*ZEB2* c.2856delG
32	11(1–12)	25(105–270)	191.9*	Nl	Nl	Nl	Nl	Nl	Nl	

Symbols and *abbreviations*: *NA* not available, *FSH* follicle stimulating hormone, *AMH* anti-Müllerian hormone, *ref* age-specific reference value, *T* testosterone, *Nl*, normal.

# Determined between day 14–90; $: age-specific AMH reference values may differ according to the commercial kits that have been used during the course of the study; *Basal testosterone value between day 14–90/**: Testosterone value after HCG stimulation (1500 U, blood sampling after 72 h).

Genomic coordinates based on build hg18 (2006), except for patient 32, where build hg19 (2009) is used.

#### Cell culture, RNA extraction and cDNA synthesis

Lymphocytes were isolated by Lymphoprep™ (STEMCELL Technologies) and cultured in RPMI medium with 10% FCS; interleukin-2 and phytohemagglutin were added. Cells were incubated at 37°C and 5% CO_2_. RNA was extracted using the RNeasy Plus Mini kit (Qiagen), followed by cDNA synthesis with the iScript™ cDNA synthesis kit (Biorad).

#### Expression analysis

Expression levels of *NR5A1* were measured through real-time quantitative PCR (rt-qPCR), using following primers: *NR5A1*-F 5′ caggagtttgtctgcctcaa 3′ and *NR5A1*-R 5′ agtggcacagggtgtagtca 3′. After *in silico* validation primers were tested using a dilution series. The experiment was done with the SsoAdvanced SYBR supermix (Bio-rad). Analysis of rt-qPCR results was done with qbase + software (Biogazelle).

The study was approved by the local medical ethical committee (Registration number B670201110608) and all parents signed a written informed consent.

## Results

### Clinical investigation

Consanguinity was present or suspected in 4/32 cases (12.5%). Another four cases had a family history of subfertility or atypical genitalia. Nine children (28.1%) were born small for gestational age (SGA), defined as a BW < −2 Standard Deviation (SD) for gestational age, with a mean BW of −2.8 SD; mean BW of children born appropriate for GA was −0.36 SD. EMS scores ranged from 2/12 to 12/12. In 6/32 children (18.7 %) dysmorphic features were noticed. Patient details are represented in Table 1.

Three out of 32 patients (P26, P28, P29) were diagnosed with KS based on clinical and hormonal data (day 14–90). Patient 26 (EMS 12) was referred for an atypically looking short penis (with bilateral descended testes). At physical examination, stretched penis length (SPL) measured 30 mm, but his penis was extremely thin and weak, reminiscent of agenesis of the corpora cavernosa, which was excluded by Magnetic Resonance Imaging (MRI) of the penile structures. Hormonal data concordant with hypogonadotropic hypogonadism (HoH) (Table 2) and MRI revealing a hypoplastic bulbus olfactorius were both consistent with a diagnosis of Kallmann syndrome. An etiological diagnosis was sought by targeted resequencing of several known KS genes (*KAL1, FGFR1, FGF8, CHD7, PROK2, PROKR2, HS6ST1, WDR11, SEMA3A, GNRH1, GNRHR, KISS1, KISS1R, TAC3* and *TACR3*); no causal mutations were identified. The second patient with KS (P28, EMS 8) presented with mild craniofacial dysmorphism (ptosis, plagiocephaly), general hypotonia, developmental delay, micropenis (SPL 15 mm) and bilateral inguinal testes. Low gonadotrophins in association with a low AMH was suggestive of HoH. Array-CGH revealed a causal hemizygous deletion on the X chromosome including the *Kallmann syndrome 1* (*KAL1)* gene, as discussed below. Patient 29 (EMS 9) was diagnosed with KS based on the presence of micropenis (SPL 21 mm) and a positive family history for KS: the father had been diagnosed with KS and was able to conceive following gonadotrophin therapy. Hormonal data confirmed HoH in the index patient. The diagnosis was supported genetically by the identification of a heterozygous *FGFR1* mutation, c.1042G > A (p.G348R), in both the patient and his father. This mutation has been described previously [23].

**Figure 1:**
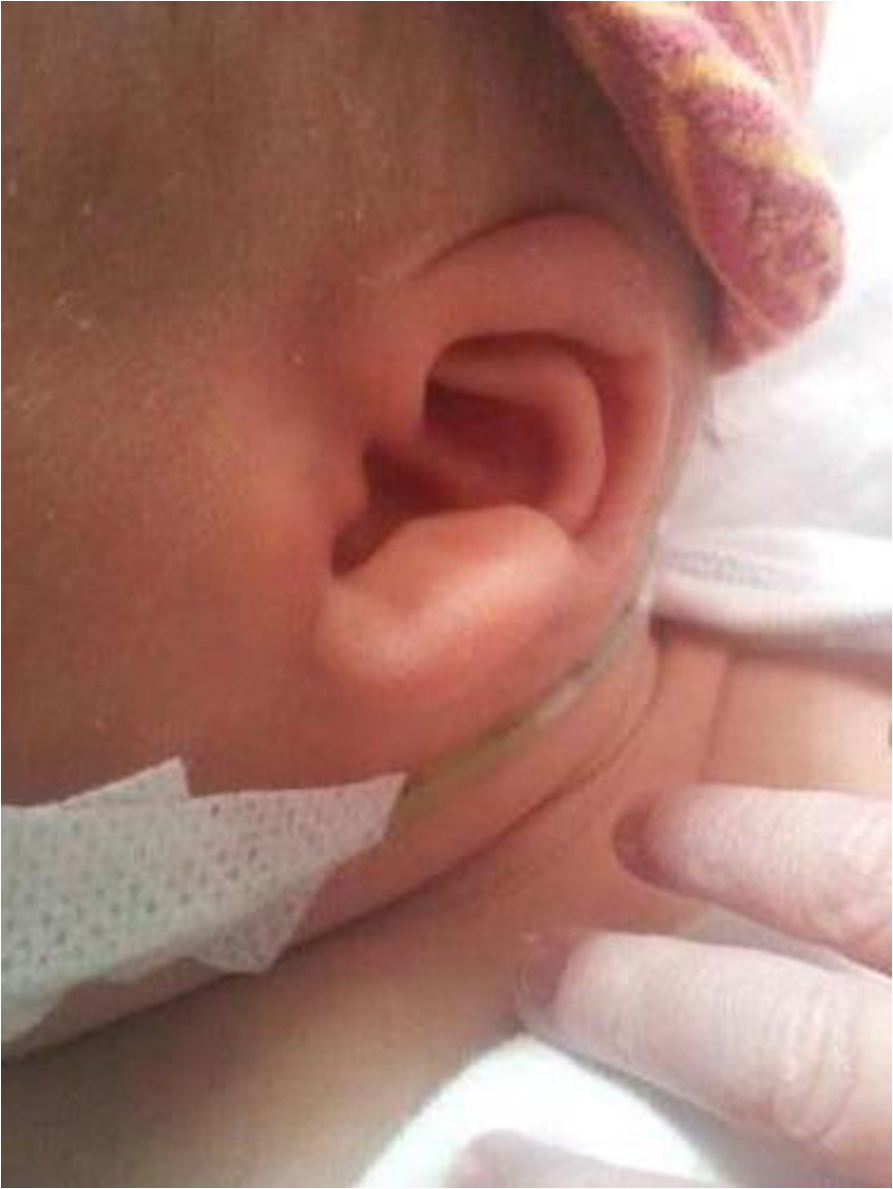
**Mowat-Wilson syndrome, facial characteristics.** The typical large and uplifted earlobes in Patient 30, who was diagnosed with Mowat-Wilson syndrome based on clinical data.

Patient 30 was diagnosed with Mowat-Wilson syndrome (MWS), he presented with typical external ear abnormalities (Figure 1), hypotonia, persistent ductus arteriosus, ventricular septum defect, facial dysmorphism, Hirschsprung disease, penoscrotal inversion and hypospadias. MWS is caused by heterozygous *de novo* mutations in *ZEB2*. Sequencing of this gene revealed a heterozygous one basepair frameshift deletion, c. 2856delG (p.Arg953Glufs*24).

### Hormonal work-up

With the exception of cases with KS, where FSH was low, serum FSH was within the reference range in all cases. AMH, representing Sertoli cell function, was low in 11/32 cases (34,3%), including 2/3 cases with KS (in the third KS case, AMH could not be determined) and 4/9 cases (44%) born SGA. Low AMH was associated with low T values (a marker for Leydig cell function) in only two cases (P3, subsequently diagnosed with a *NR5A1* mutation and P26, with KS). Two of three patients with *NR5A1* mutations had an AMH value within the reference for age. Ratios of T/A and T/DHT were determined to identify possible cases of (dihydro)testosterone biosynthesis disorders. The T/A ratio, measured during mini-puberty was suggestive of 17β-HSD deficiency in two patients (case 1: T/A ratio 0,19; case 2: T/A ratio 0,52) and after HCG stimulation in one case (case 17: T/A ratio 0,08) [21,22]. *HSD17B3* sequencing was performed in all three cases but revealed no causal mutations. In patient 2 a heterozygous missense variant was identified, c. 866G > A (p.Gly289Asp), although mutation prediction programs indicated this variant to be tolerated. In patient 24, a T/DHT ratio of 10,8 was found at basal sampling during mini-puberty but *SRD5A2* sequencing revealed no mutations.

### Genetic work-up

Array-CGH was done in 23/32 patients to screen for larger genomic rearrangements. In 10 of them, CNVs were identified as shown in Table 2. Seven of these rearrangements were maternally inherited, making their clinical significance questionable. In patient 11, we identified a partial chromosome 9 deletion (9p24.3), encompassing the *Doublesex and Mab3 related transcription factor 1* (*DMRT1*) gene. In patient 28, a deletion was found on the X chromosome (Xp22.31-Xp22.32). This region includes the STS region and the genes *KAL1* and *Neuroligin 4, X-linked* (*NLGN4X*). This deletion was also present in the patient’s mother. In addition, we performed MLPA for 23/32 patients to screen for deletions and/or duplications on the exon level, however no additional CNVs were identified.

*AR* (20/32) and *WT1* (22/32) sequencing did not reveal any mutations. *NR5A1* sequencing was done in 26/32 patients, leading to the identification of three novel mutations, which will be discussed below. In cases with serum AMH below the reference value for age (8/32), suggestive of gonadal dysgenesis, *SRY* was sequenced, however no mutations were found.

### Identification of three novel *NR5A1* mutations

**Figure 2:**
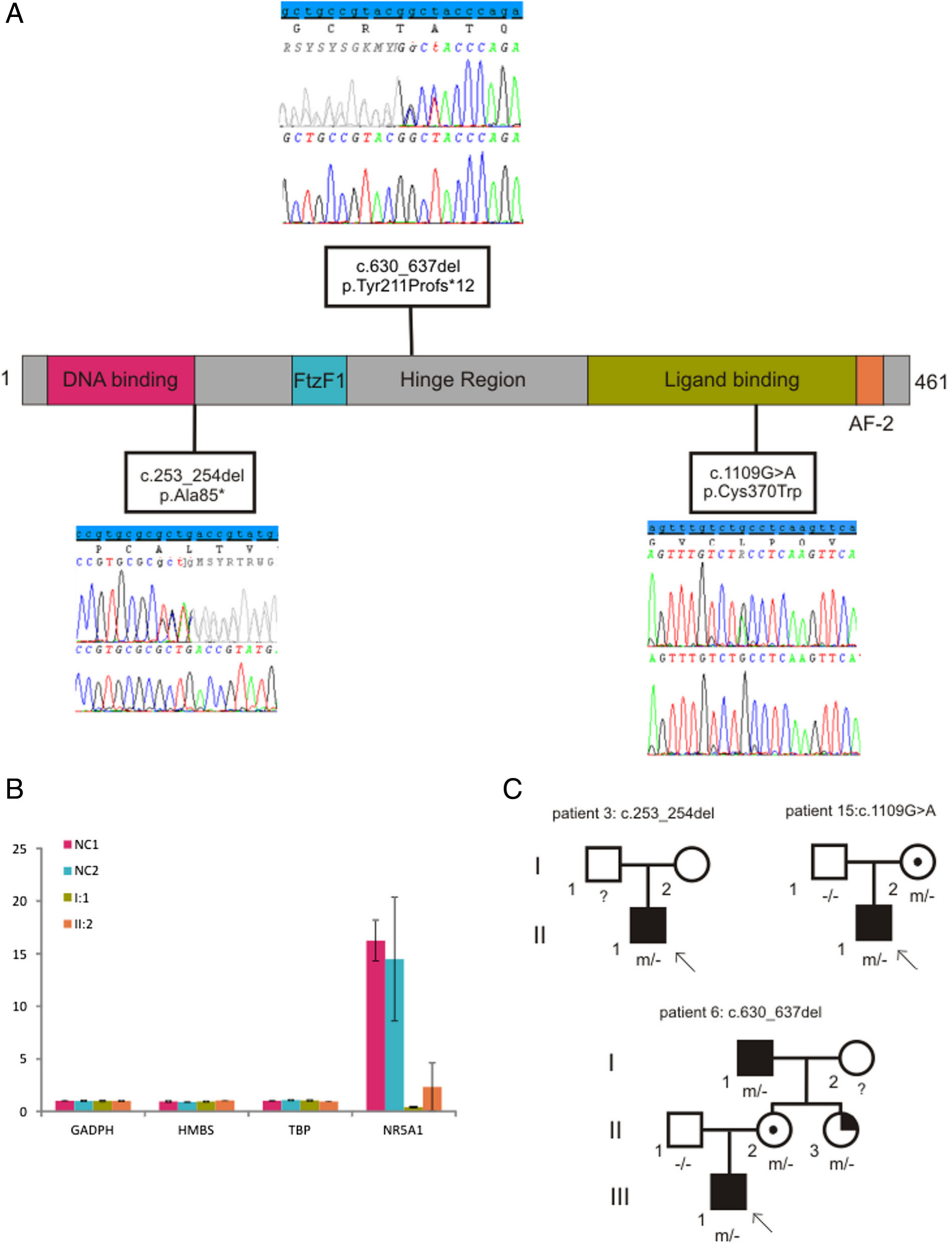
**Three novel** ***NR5A1*** **mutations. (A)** Schematic overview of the positions of the mutations and electropherograms. **(B)** RT-qPCR showed a lower *NR5A1* expression in the maternal grandfather of the index patient (I:1), and in the mother of the index patient (II:2). We did not include the index case in this experiment as no fresh blood could be collected. Two negative control samples (NC) without the mutation were included for comparison. To exclude technical variations, expression of the reference genes *GADPH, HMBS* and *TBP* were also measured, showing stable expression in all patients. **(C)** Pedigrees for the patients with a *NR5A1* mutation. The genotype of the analysed individuals is shown under their symbol. Full black squares indicate affected males with hypospadias, partially black circles indicate females with POF and circles with a black dot correspond with asymptomatic carrier females.

*NR5A1* sequencing revealed three novel mutations (Figure 2A). In patient 3 a heterozygous frameshift deletion was identified: c.253_254del, resulting in a premature stopcodon (p.Ala85*). No other family members were available for segregation analysis. A second heterozygous frameshift deletion of 8 bp was identified in patient 6, c.630_637del, (p.Tyr211Profs*12). Rt-qPCR in the patient’s lymphoblasts indeed showed a lower expression of *NR5A1* mRNA (Figure 2B). Segregation analysis indicated that this mutation was present in (1) the asymptomatic patient’s mother, (2) maternal aunt, who had been diagnosed with POF at the age of 35, and (3) grandfather, who had been operated for proximal hypospadias, but spontaneously fathered two children (pedigrees in Figure 2C). The third mutation was found in patient 15, c.1109 G > A, (p.Cys370Trp). This mutation was predicted to have a deleterious effect on protein function according to several prediction programs (SIFT, Polyphen and MutationTaster). The affected amino acid is located in the ligand-binding domain and is highly conserved (up to zebrafish). Segregation analysis revealed that the mutation was present in the patient’s mother, who had no symptoms of POF at the age of 24.

## Discussion

**Figure 3:**
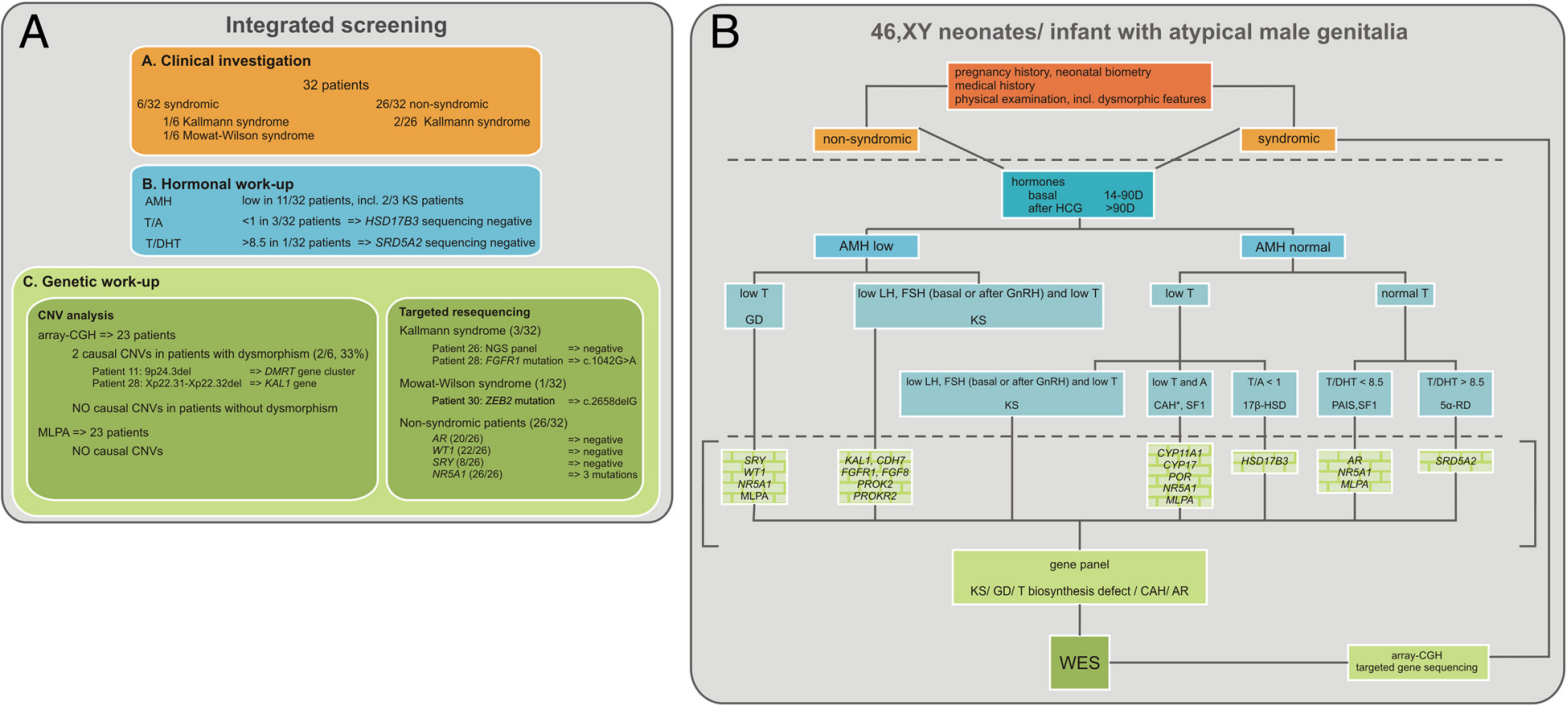
**Overview of the integrated investigation approach. (A)** Results in the 46,XY undervirilization cohort. Clinical and hormonal investigation was sufficient to suspect a diagnosis in 4/32 cases. For two Kallmann syndrome patients the diagnosis was genetically confirmed, as shown in the CNV analysis and targeted resequencing boxes. A *ZEB2* mutation was identified in the Mowat-Wilson syndrome patient. Subsequently a genetic work-up was performed for the remaining patients, guided by hormonal results. Sequencing of *HSD17B3* and *SRD5A2* in patients with a possible testosterone biosynthesis disorder did not reveal mutations. Genetic screening consisting of array-CGH, DSD MLPA and sequential gene-by-gene sequencing led to the identification of two causal CNVs (of which one KS, see above) and three novel *NR5A1* mutations, respectively. **(B)** Suggested clinical algorithm for the investigation of 46,XY male neonates or infants referred for atypical genitalia. Upper section (orange): clinical investigation, including pregnancy history, medical history and physical examination, enables categorization in cases with and without syndromic features. . Mid-section (blue): In all cases, clinical investigation should be followed by a hormonal work-up, which in turn can be suggestive of gonadal dysgenesis (GD), disorders of the steroid hormone biosynthesis pathway and/or rare forms of CAH (*:Only forms characterized by defective androgen production are implicated here), partial androgen receptor defects or KS. Insights in hormone levels can guide selection of target candidate genes. Lower section (green): After thorough evaluation of clinical and hormonal data, a decision can be made to sequence specific gene panels or to proceed to clinical whole exome sequencing to identify the underlying molecular cause and thereby support the clinical diagnosis. The boxes between brackets (with squared filling) represent single gene tests which can be replaced be the aforementioned gene panels In cases with syndromic features, array-CGH is still a recommended method to identify CNVs.

To gain insight into the appropriateness and diagnostic yield of a systematic integrated work-up in 46,XY undervirilized cases who are sex-assigned male, we used a standardized screening panel in a series of 32 cases referred to our DSD clinic. An overview of the approach is shown in Figure 3A. Difficulties in blood collection in newborns and infants made it impossible to perform the complete screening in every case, resulting in missing data. Low EMS scores (EMS < 7, n = 17) did not yield a higher diagnostic success as compared to higher EMS scores (EMS ≥ 7, n = 15). As reported earlier, no causal genetic variations were identified in children born SGA (n = 9) in our series [24].

### Clinical investigation and hormonal data were sufficient to diagnose Kallmann syndrome and Mowat-Wilson syndrome in respectively three and one patients

Familial, hormonal and/or phenotypic data were sufficient to suspect KS in three patients (P26, 28 and 29) and MWS in patient 30. As suggested in a paper by Grumbach et al. our study confirms that in boys, the period of physiological gonadotrophin surge (the so-called “mini-puberty”) represents a unique opportunity to diagnose KS early in cases with a suggestive phenotype (micropenis +/− cryptorchidism in the absence of hypospadias) [25]. In these patients a targeted approach to identify the underlying molecular cause was used. Here, we ended up with a higher diagnostic success rate, the molecular cause was identified in 75% (3/4) of patients.

### Despite suggestive hormonal results, we could not identify any *HSD17B3*, *SRD5A2* or *SRY* mutations

Accumulation of A or T due to 17β-HSD deficiency or 5α-reductase deficiency respectively may lead to markedly low T/A (in case of 17β-HSD deficiency) or elevated T/DHT (in case of 5α-reductase deficiency) ratios. In contrast to previous reports, sequencing of the *HSD17B3* and *SRD5A2* genes in cases with aberrant T/A and T/DHT ratios respectively revealed no mutations [21,22,26,27]. However, for practical reasons, stimulated A and T values, which are generally considered more accurate than basal values during mini-puberty, had been obtained in only one of three patients with T/A < 1. Another possible explanation might be the different detection methods used for the various androgens (Radioimmunoassay for A versus LC/MSMS for T). Simultaneous detection of A, T and DHT by LC/MSMS, as described recently, is expected to be more reliable but is not routinely available yet [28]. Low serum AMH has been reported previously in infants with KS [3,29], and has been attributed to a lack of FSH-driven stimulus [30,31].

In all cases in which serum AMH was below the age-specific reference values (n = 10), sequencing of *SRY* was performed but revealed no mutations, confirming that *SRY* mutations are a relatively rare cause of 46,XY partial gonadal dysgenesis in contrast to 46,XY complete gonadal dysgenesis, where *SRY* mutations are thought to account for up to 15% of cases [3,32].

### Genetic screening: targeted resequencing and copy number analysis

Following a number of recent reports in which *NR5A1, AR* and *WT1* mutations and CNVs have been identified as the cause of isolated hypospadias and/or cryptorchidism [10,12,33-39], a standardized genetic screening protocol was applied to identify the underlying genetic cause of the observed atypical genital development in all cases where clinical and hormonal data did not suggest a specific diagnosis, irrespective of the EMS scores. The screening consisted of array-CGH, MLPA and *SRY*-specific FISH to screen for genomic rearrangements, and sequencing of the *AR*, *WT1* and *NR5A1* genes.

#### Array-CGH is a valuable diagnostic tool in 46,XY undervirilization newborns with dysmorphic features and allowed the identification of two causal CNVs in our cohort

Array-CGH was used to screen for larger genomic rearrangements and led to the identification of two deletions with clinical significance, both found in syndromic patients. Patient 11 (EMS = 3) presented with penoscrotal hypospadias and transposition. Besides these genital characteristics, this patient also showed macrocephaly, facial dysmorphism and developmental delay. Hormonal results revealed normal T levels, AMH was not available; array-CGH revealed a partial chromosome 9 deletion, encompassing the *DMRT* gene cluster. These genes encode transcriptional regulators involved in sex development, and monosomy of the distal part of chromosome 9p, mostly *DMRT1*, has been associated with 46,XY DSD in several cases [40,41]. Patient 28 (EMS = 8) showed symptoms of KS. Other phenotypic characteristics included: X-linked ichthyosis, hypotonia, recurrent kidney stones and developmental delay. Liver function tests showed abnormal results, of hitherto unknown etiology. In this patient a part of the X-chromosome, including the genes *KAL1* and *NLGN4X,* was deleted. *KAL1* deletions or mutations are an established cause of X-linked KS and can explain the genital phenotype seen in this patient [42]. *NLGN4X*, has been associated with X-linked mental retardation and X-linked autism spectrum disorders [43], and might explain the observed developmental delay. Previously, a link between KS, ichthyosis and Xp deletions has been described by Bick et al. [44]. No evident association could be found between the identified deletion and the elevated liver enzymes and recurrent kidney stones. This deletion was inherited from the mother, who had mild mental delay but no symptoms of KS. This deletion is therefore characterized by incomplete penetrance.

In total, array-CGH revealed 10 CNVs in 22 patients, seven of them were inherited from the mother; making their clinical relevance questionable. Array-CGH resulted in a definite genetic diagnosis in 2/22 patients, (9%). When only considering the syndromic cases, arrayCGH renders a diagnostic yield of 2/6 patients (33%). Although our series is small we can conclude that array-CGH is a valuable diagnostic tool in 46,XY DSD with associated dysmorphic features however larger patient groups should be investigated to make more definite conclusions. Because of the limited resolution of array-CGH, we performed MLPA to screen for deletions or duplications on the exon level for *SOX9, NR5A1, WNT4* and *NR0B1.* In total 23 patients were screened, however no additional CNVs were identified. Likewise, FISH analysis of *SRY* could not reveal any deletions. Although the mutation uptake of targeted CNV detection (MLPA) was limited in our cohort, it still remains an important addition to a genetic work-up of 46,XY undervirilized or 46,XY DSD patients. Different reports showed *NR5A1* microdeletions as a cause of both 46,XY DSD and POF [16,45]

#### We identified three novel NR5A1 mutations, one of them was present in an affected male with preserved fertility

Recently Kohler et al. reported a *WT1* mutation rate of 7.5% in children with severe hypospadias and Wang et al. identified *AR* mutations in 6.6% of their patient cohort with isolated hypospadias and micropenis, indicating a role for both *WT1* and *AR* in minor forms of undervirilization [4,34]. Sanger sequencing of *AR* and *WT1* was done in respectively 20 and 22 patients of our cohort. In contrast to these series, no significant sequence changes in these genes were identified. The relatively high frequency in previous cohorts might be attributed to a selection bias. Therefore, we conclude that the incidence of mutations in *AR* and *WT1* mutations is probably overestimated in patients with milder forms of undervirilization. On the other hand, *NR5A1* was sequenced in 26 patients and revealed mutations in three of them (11.5%). This is in line with other series, where mutations were identified in approximately 15% of patients. In our cohort, two frameshift mutations and one missense mutation were identified. The missense mutation, c.1109G > A, found in patient 15 (EMS = 3), targets an amino acid in the functionally important ligand binding domain (p.Cys370Trp) and is predicted to alter protein function (SIFT, Polyphen, MutationTaster). This mutation was also found in the patient’s mother. In addition to causing 46,XY DSD, *NR5A1* mutations are a known cause of premature ovarian failure (POF) [46]. The patient’s mother had regular menses at the age of 30, however she is at risk for developing POF. The first frameshift mutation (patient 3), c.253_254del induces a premature stop codon at position 85 (p.Ala85*). There were no additional family members available for segregation analysis. The second frameshift mutation (patient 6), c. 630_637del, also leads to a premature stop codon (p. Tyr211Profs12*). This mutation was also present in the mother of the patient, a maternal aunt and the maternal grandfather. The aunt had recently been diagnosed with POF at the age of 35 years and underwent several in vitro fertilization (IVF) cycles, the patient’s mother (age 39) declared to have regular menses. Interestingly, the grandfather had been treated for hypospadias as a child. Preserved fertility in males with *NR5A1* mutations has only exceptionally been reported so far [47,48]. These findings support the extreme intra-familial variability seen with *NR5A1* mutations. At the moment the mechanism behind this phenotypic variability and incomplete penetrance resulting from *NR5A1* mutations remains elusive; they likely result from the effects of multiple genetic variations (modifiers) and/or their interactions with environmental factors. Variable expressivity, reduced penetrance and even more complex inheritance patterns such as digenic models have been reported in other developmental conditions such as Kallmann syndrome and may be explained in part by the overall ‘mutational load’ in different genes playing a role in common signaling pathways [49-51].

### The integrated story: clinical, hormonal and genetic data

Taken together, in spite of extensive clinical, hormonal and genetic screening, the molecular cause of 46,XY atypical male genital development could only be identified in seven out of 32 patients (21.8%). When comparing the diagnostic success rate between patients with low (<7, n = 17) or high (≥7, n = 15) EMS scores, we identified the underlying molecular defect in respectively three and four patients, leading to a diagnostic success rate of respectively 17.6% and 26.5% for patients with low versus higher EMS scores, suggesting that the decision to perform a detailed diagnostic work-up in 46,XY patients with atypical genitalia should not be based on the severity of the phenotype alone. Array-CGH revealed the causal CNV in two out of six syndromic patients, leading to a diagnostic yield of 33% in patients with additional phenotypic characteristics. When we included non-syndromic cases, the success rate drops to 9%, indicating that array-CGH is still an appropriate diagnostic tool in syndromic forms of 46,XY DSD, but is less efficient in non-syndromic cases. Sequencing of *AR*, *WT1* and *SRY* did not reveal any mutations. Besides the low diagnostic yield of this sequential sequencing approach, cost and time efficiency should be considered. Sanger sequencing has an average cost of $2400 per million bases, whereas the emerging next generation sequencing technologies (NGS) are much cheaper. With the Illumina platform, there is only a $0.07 sequencing cost per million bases (number based on Hiseq2000) [52]. The next step in the diagnostic work-up of 46,XY boys wit atypical genitalia should be the implementation of targeted NGS panels covering clinically relevant genes with a known role in sex development and steroid biosynthesis pathways. A flexible and automated NGS workflow used for targeted resequencing of disease gene panels has been reported by us and allows parallel and cost-effective analysis of a sizeable number of genes in a clinical setting (De Leeneer et al. Human Mutation provisionally accepted). While this approach seems to be very useful in some heterogeneous disorders, their clinical utility in 46,XY DSD is debatable, since the known disease genes in these phenotypes only account for 20–40% of patients. Therefore we anticipate that whole exome sequencing (WES), which is increasingly put forward as a clinical diagnostic test in genetically heterogeneous disorders [53,54], will gain importance in the diagnostic work-up of 46,XY DSD, both in a clinical and research context. However, in cases where associated phenotypic characteristics or cases where clinical and hormonal data suggest a specific gene defect, it remains advisable to perform targeted resequencing of the specific disease gene(s).

## Conclusion

In this study we examined a large consecutive cohort of undervirilized 46,XY neonates and infants. Following this protocol we were able to genetically diagnose 19% of non-syndromic patients and one third of the syndromic cases. There was no significant difference between the diagnostic success rate in patients with low EMS compared to higher EMS. In syndromic cases, array-CGH had a high diagnostic yield. Serial gene screening resulted in several novel *NR5A1* mutations, although the overall diagnostic yield was rather low. Interestingly, we identified a novel *NR5A1* mutation that was also present in a related male with preserved fertility, which has only exceptionally been reported. Given the low diagnostic yield of the sequential approach, parallel screening technologies such as targeted resequencing of clinically relevant disease genes and WES will be a preferred choice in future screening protocols. However, in cases where associated phenotypes are present, a more targeted approach remains the preferential strategy.

## Additional file

Additional file 1: Table S1. Overview of the primer sequences for *NR5A1* (NM_004959.4), *HSD17B3* (NM_000197.1) and *SRD5A2* (NM_000348.3).

## Abbreviations

A: Androstenedione
AMH: Anti-Müllerian hormone
Array-CGH: Array- comparative genomic hybridization
CNV: Copy number variant
DSD: Disorders of sex development
DHT: Dihydrotestosterone
EMS: External masculinization score
FISH: Fluorescent in-situ hybridization
FSH: Follicle stimulating hormone
HoH: Hypogonadotropic hypogonadism
IVF: In vitro fertilization
KS: Kallmann syndrome
LH: Luteinizing hormone
MLPA: Multiplex ligation dependent probe amplification
MWS: Mowat-Wilson syndrome
POF: Premature ovarian failure
Rt-qPCR: Real-time quantitative PCR
SD: Standard deviation
UTR: Untranslated regions
WES: Whole exome sequencing
