# Supplementary material for: Extensive clinical, hormonal and genetic screening in a large consecutive series of 46,XY neonates and infants with atypical sexual development
Source: Orphanet J Rare Dis. 2014 Dec 14;9:209. doi: 10.1186/s13023-014-0209-2 (PMC4271496; doi:10.1186/s13023-014-0209-2)
Supplement: Additional file 1: Table S1. — Overview of the primer sequences for NR5A1 (NM_004959.4), HSD17B3 (NM_000197.1) and SRD5A2 (NM_000348.3). [file 13023_2014_209_MOESM1_ESM.docx]

## Supplemental data

Additional file 1: Table S1: overview of the primersequences for *NR5A1* (NM_004959.4), *HSD17B3* (NM_000197.1) and *SRD5A2* (NM_000348.3)

| *NR5A1*- ENSE00001460969 | gggcacagagaggggzttac | atcttgcagctctggctctc |
| --- | --- | --- |
| *NR5A1*-ENSE00000726938 | aaggtgtccggctaccacta | actatcccctcagcccctct |
| *NR5A1*-ENSE00000726937-A | ttgtttggaaaggatctgtgg | agagaagggctctgggtagc |
| *NR5A1*-ENSE00000726937-B | tgagtacccggagccttatg | aaggatggccctatccaaag |
| *NR5A1*-ENSE00000726936 | atctgggtagatgggcacag | gtggggaaagggctgataat |
| *NR5A1*-ENSE00000726934 | acctgcacctccaatccat | tgagtaagggaggggtcctt |
| *NR5A1*-ENSE00001230484 | caatgcccatgtctttgatg | tcccaaacacacagtgtcaga |
| *HSD17B3-*ENSE00000895687 | cccctgacttgatggaacac | tgggtgacagagcaagtgtc |
| *HSD17B3*-ENSE00000895686 | tttagtgaggggcttcaagg | aaaagagatccagggagtgaga |
| *HSD17B3*-ENSE00000895685 | gggttgtgttgttcctagcc | gccagtgtgcactaaagctg |
| *HSD17B3*-ENSE00003672420 | ggaaccaccattcaccaatc | gcctctagaagaagtgtgctttg |
| *HSD17B3*-ENSE00003611256 | cccagtttcctcaccgttac | gaaagggggcctcaatacat |
| *HSD17B3*-ENSE00003600030 | gctggaaggcagttatgagc | aacaagacaattacaagaccacga |
| *HSD17B3*- ENSE00003573360 | gtctaggttggctcctgtgg | tcaccgtgtgctttcatcat |
| *HSD17B3*-ENSE00003506291 | gggaaggtaggggaagttca | agtagctcagccctgcacat |
| *HSD17B3*-ENSE00003652031 | accctgtgcagctgaaactc | cctgttgaccacacatgagc |
| *HSD17B3*-ENSE00003604146 | aagagaagggtcgggaaatg | tcgccacatagtccttgtacc |
| *HSD17B3-*ENSE00003636684 | tttggcgtctgagaaaacct | cacaccaatttcctgggtct |
| *SRD5A2*- ENSE00001559957 | gcgggcgaactaagaagg | tcaactctctagcgtccaagc |
| *SRD5A2*- ENSE00003529006 | gttaaggcgaaatggcaga | gggaagatgggatcattacg |
| *SRD5A2*- ENSE00003475002 | ccactttctgccacgtctta | caggggaagtcaagagcaag |
| *SRD5A2*- ENSE00003544207 | gctgcctttgtgtattttgga | gcccagcaagtcagaatatg |
| *SRD5A2*- ENSE00001549980-A | aagaatcataagtgaccatcgaaa | aagtactgccttcaagtcaaaaa |
| *SRD5A2*- ENSE00001549980-B | atgtctttcaccgggcaat | tttgttttacactactcattatttgga |
| *SRD5A2*- ENSE00001549980-C | catgaacctgggtggcttat | accccttcacaagagtttgc |
| *SRD5A2*- ENSE00001549980-D | ttaaaaatcaggagattccttca | acagggtgaatgggaatgag |
